# The Effects of Maternal Metformin Treatment on Late Prenatal and Early Postnatal Development of the Offspring Are Modulated by Sex

**DOI:** 10.3390/ph13110363

**Published:** 2020-11-04

**Authors:** Consolacion Garcia-Contreras, Marta Vazquez-Gomez, José Luis Pesantez-Pacheco, Ana Heras-Molina, Teresa Encinas, Susana Astiz, Antonio Gonzalez-Bulnes

**Affiliations:** 1 SGIT-INIA, Ctra. De La Coruña Km. 7,5, 29040 Madrid, Spain; congarcon@gmail.com (C.G.-C.); jose.pesantez@ucuenca.edu.ec (J.L.P.-P.); delasheras.ana@inia.es (A.H.-M.); astiz.susana@inia.es (S.A.); 2 Faculty of Veterinary Medicine, UCM, Ciudad Universitaria s/n., 28040 Madrid, Spain; martavazgomez@gmail.com (M.V.-G.); tencinas@vet.ucm.es (T.E.); 3 Facultat de Veterinària, Universitat Autònoma de Barcelona. Edifici V, Trav. dels Turons, 08193 Bellaterra, Spain; 4 Faculty of Agricultural Sciences, School of Veterinary Medicine and Zootechnics, University of Cuenca, Avda. Doce de Octubre, 010220 Cuenca, Ecuador

**Keywords:** intrauterine growth restriction, metformin, pregnancy, swine model

## Abstract

Metformin is currently used to improve pregnancy outcome in women affected by polycystic ovary syndrome (PCOS) or diabetes. However, metformin may also be useful in pregnancies at risk of intrauterine growth restriction (IUGR) since it improves placental efficiency and the fetuses’ developmental competence. There is no data on the duration of the effect of this treatment from the prenatal up to the postnatal stages. Therefore, the present trial aimed at determining the impact of metformin treatment on the offspring neonatal traits and early postnatal development (i.e., during lactation) using an in vivo swine model. The results support that maternal metformin treatment during pregnancy induces protective changes in body shape and composition of the progeny (i.e., larger head size and body length at birth and higher total viscera weight at weaning). However, there were also major effects of the offspring sex (smaller corpulence in females and lower relative weight of main viscerae in males), which should be considered for further preclinical studies and when even the current clinical application in women affected by PCOS or diabetes is implemented.

## 1. Introduction

Intrauterine growth restriction (IUGR), the failure of a fetus to reach its full genetic growth potential, affects between 6% and 17% of total human pregnancies; a range depending on environmental and socioeconomic factors. IUGR results in small-for-gestational-age (SGA or low-birth-weight, LBW) offspring, with SGA being the second leading cause of infant mortality and morbidity after premature birth. IUGR directly affects the life-quality and well-being of many individuals and causes a huge financial burden to the public health care systems [1].

In consequence, IUGR has been the focus of intense research during the last 40 years, but mainly during the last ten years [2] as the traditional causes for IUGR (maternal malnutrition and hypoxia in developing areas and extreme environments) are reinforced by a contemporary increase in the incidence of “placental insufficiency” in both developing and developed countries [3]. The term “placental insufficiency” defines a deficiency in the placental development and function, causing a shortage of the transfer of nutrients and oxygen to the fetus. This condition is currently estimated to be the cause of around 60% of IUGR cases [4]. Moreover, placental insufficiency is a rising problem, since it is linked to many contemporary factors (delay in childbearing age, inadequate lifestyle, stress, sedentarism, pollution, alcohol and tobacco intake, obesity, diabetes or preeclampsia [5]).

The current magnitude and the expected future increment of IUGR incidence make necessary to increase the research on diagnostic, preventive, and therapeutic approaches. Preventive and therapeutic strategies are mainly based, to date, on lifestyle and diet changes, since there are no therapeutic actions with proven validity and the final solution is the induction of a preterm delivery. In fact, around 40% of premature births, which are the leading cause for infant mortality and morbidity, is related to the medical indication to induce birth before the 34th week of gestation, due to IUGR [6]. Therefore, research for therapeutic tools focuses on the improvement of placental development and functionality by either pharmacological treatments (e.g., aspirin or sildenafil citrate) or nutritional supplementation (e.g., amino acids, vitamins favoring protein synthesis or antioxidant status, and other antioxidants such as polyphenols). Such research cannot be carried out in human beings, so the use of animal models (either rodents or large animals) is unavoidable [7].

Our group has approached, using a well-proven swine IUGR model [8,9], a distinctive pharmacologic alternative: the use of metformin. Metformin is a drug widely used for the treatment and prevention of diabetes [10], and it is currently tested to prevent large-for-gestational-age (LGA) offspring in pregnant mothers with diabetes [11,12], the opposite scenario to IUGR and SGA offspring. However, metformin favors the transfer and uptake of glucose to peripheral tissues [13] without inducing maternal hypoglycemia [14]. Our hypothesis was that metformin treatment in IUGR compromised pregnancies might favor the uptake of glucose by the placenta and fetus and, consequently, improve fetal development. We found that the maternal treatment with metformin, despite no significant effects on fetal body mass, favored placental development and fetal viscera growth (mainly brain, liver, kidneys, spleen, and adrenal glands) [15]. These organs are pivotal for the survival and development of the newborns, with these findings suggesting a beneficial effect of the metformin treatment on the fetuses’ developmental competence and, afterwards, neonates.

These results are of high value for human medicine in case of maternal malnutrition, frequent in developing areas. Metformin is a cheap drug easily available which seems to improve placental development, in case of placental deficiency. However, there are no data on the duration of these effects up to postnatal stages of the offspring or on the safety use of the drug during pregnancy [16]. Thus, the present trial aimed at determining the effects of the maternal metformin therapy on neonatal traits and early postnatal development (i.e.: during lactation) of the offspring in mammals.

## 2. Results

### 2.1. Effects of Maternal Metformin Treatment on Late Prenatal Development and Neonatal Features

The mean litter size was similar in both control and metformin-treated groups (7.8 ± 0.6 piglets/litter in the Group Control, Group C, and 8.0 ± 0.7 piglets/litter in the treated group, Group METF). The sex ratio of piglets was close to 1:1 in both groups, with 21 female and 26 male piglets in group C (44.7% and 55.3%, respectively) and 38 female and 34 male piglets in the group METF (52.7% and 47.3%, respectively). 

There was no disparity in the mean birthweight between piglets between the groups (Table 1), but there were significant birth-size differences. Piglets in the group METF, independently of sex, showed a larger head size (in terms of higher values for both occipito-nasal length and biparietal diameter; *p* < 0.0005 and *p* < 0.005, respectively, with a higher ratio occipito-nasal to length/biparietal diameter; *p* < 0.05) and a longer body length (*p* < 0.05). Conversely, differences in corpulence were determined by an interaction sex*treatment. In this sense, METF males had similar thoracic and abdominal circumferences to those from control males, while female piglets in the group METF showed smaller thoracic and abdominal circumferences than C females (*p* < 0.005 and *p* < 0.05, respectively). A within-group comparison showed that body weight and size were similar between females and males in the group C (excepting a trend for lower body length in males, *p* = 0.06). Males in the group METF showed a trend for a higher body weight (*p* = 0.06) and higher values for occipito-nasal length and thoracic circumference (*p* < 0.05 for both) than their female littermates.

### 2.2. Effects of Maternal Metformin Treatment on Early Postnatal Development and Body Composition

**Figure 1:**
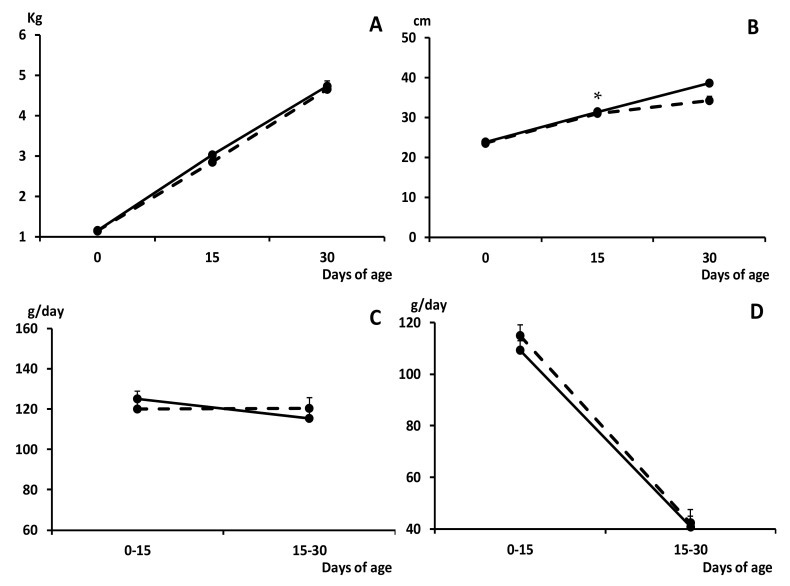
Change over time in mean values (±S.E.M.) of body weight and length (panels (**A**) and (**B**), respectively), average daily weight gain (ADWG; panel (**C**)) and fractional growth rate (FGR; panel (**D**)), during the early postnatal development (0 to 30 days old) in piglets from control (group C; black dots with discontinuous lines) and sows treated with metformin during pregnancy (group METF; black dots with continuous line). Asterisk denotes significant differences between groups (* *p* < 0.05).

The growth of the piglets during the suckling period was similar in both groups, without any difference in average daily weight gain (ADWG) and fractional growth rate (FGR), and therefore in body weight, during this stage (Figure 1).

**Figure 2:**
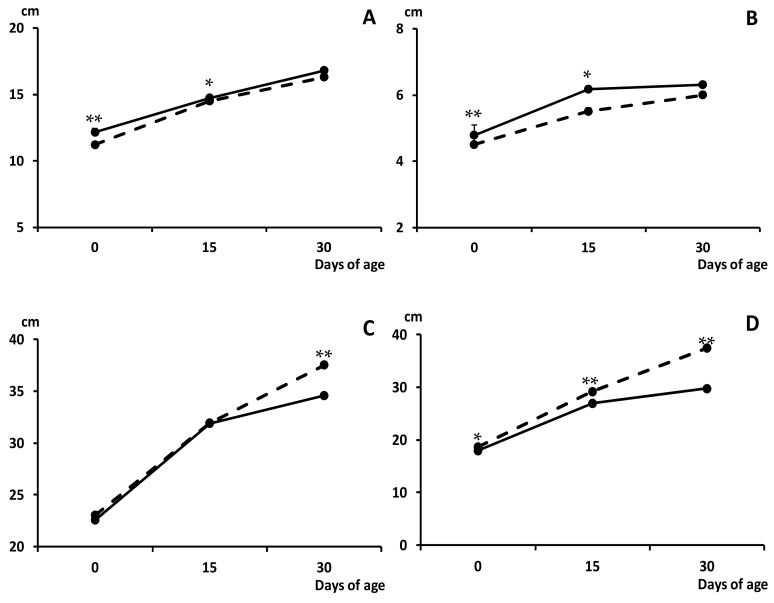
Change over time in mean values (±S.E.M.) of the occipito-nasal length (panel (**A**)), biparietal diameter (panel (**B**)) and thoracic and abdominal circumferences (panels (**C**) and (**D**), respectively) during the early postnatal development (0 to 30 days old) in piglets from control (group C; black dots with discontinuous lines) and sows treated with metformin during pregnancy (group METF; black dots with continuous line). Asterisks denote significant differences between groups (* *p* < 0.05; ** *p* < 0.01).

The higher values for occipito-nasal length and biparietal diameter found at birth in METF piglets than in C counterparts remained at 15 days old (*p* < 0.05 for both; Figure 2). Female piglets in the group METF continued showing smaller thoracic and abdominal circumferences than C females at the age of both 15 (*p* = 0.06 and *p* < 0.0005, respectively) and 30 days (*p* < 0.05 and *p* < 0.0005, respectively). Smaller thoracic and abdominal circumferences in METF males than in C males were also found at 30 days old (*p* < 0.0005 and *p* < 0.005, respectively). 

On the other hand, at weaning, METF piglets showed a higher muscle development, as determined by assessing the loin diameter (11.4 ± 0.02 vs. 9.8 ± 0.021 mm for group C; *p* < 0.0005), and subcutaneous fat accumulation, as determined by evaluating the back-fat depth (5.1 ± 0.01 vs. 4.0 ± 0.01 mm for group C; *p* < 0.0005). These differences were independent of sex.

At weaning, there were no significant differences between groups C and METF in the absolute body weight or in the absolute weight of head, carcass, and viscerae (Table 2), excepting a significant lower weight of the kidney in METF males. However, there was a trend for a higher absolute weight of total viscerae in the group METF, which resulted in a higher relative viscerae-to-body weight ratio than in the group C (*p* = 0.06 and *p* < 0.0005, respectively). The comparison of these traits between males and females within groups showed no differences in METF piglets, but the females of the group C showed a lower relative weight of the carcass and a higher relative weight of viscerae than their male group C counterparts (*p* < 0.01 and *p* < 0.05, respectively).

These differences in the weight of total viscerae influenced the assessment of the individual relative weights of the different thoracic and abdominal organs (heart, lungs, liver, intestine, kidney, spleen, pancreas, and adrenal glands). In brief, there were no significant differences in the absolute weights of the different viscerae between treatments. There were no sex-related differences within the group METF, but males in group C showed a higher absolute weight of spleen and adrenal glands than their female counterparts (*p* < 0.05 and *p* < 0.01, respectively). On the other hand, the analysis of differences in the relative weights of different organs between treatment groups showed determinant sex-related effects. The comparison of the weight of the different organs to total viscera weight showed no significant differences between C and METF females, despite numerically higher values in treated METF females. Conversely, when compared to C males, METF males had lower relative weights of kidneys (*p* < 0.0005), liver, lungs, pancreas, and spleen (*p* < 0.005 for all) and adrenal glands (*p* < 0.05) and higher relative weight of the intestine (*p* < 0.005).

## 3. Discussion

The results obtained in the present study evidence that metformin treatment during pregnancy induces a change in body shape and composition of the progeny and address a prominent role of the offspring sex on its response to such therapies.

Maternal metformin treatment during pregnancy did not induce significant differences in birthweight but in body shape, similarly to our previous study performed during the last days of pregnancy [15]. In brief, offspring from metformin-treated pregnancies had significantly higher values for head size (both when assessing occipito-nasal length and biparietal diameter) and body length. These results are highly interesting since recent studies addressed that head profile (size, length and width) and body shape (body length and width) at birth are more predictive of the postnatal development in SGA piglets than the proper birthweight [17,18].

The head profile allows discrimination between piglets that have suffered severe IUGR and piglets that have been less affected and have been born small for gestational age (e.g., proportionally small [19]). In this sense, a dolphin-like forehead (a small and short but wide head) in SGA piglets is clearly indicative of severe IUGR with an adaptive brain sparing effect [18,20]. In our study, the head was significantly larger and longer in metformin-treated piglets than in control ones, suggesting an alleviation of IUGR effects. A similar effect was described in human pregnancies affected by polycystic ovarian syndrome (PCOS) and therefore treated with metformin [21,22]. However, there is still unclear the association between metformin and increases in head size and how these changes might affect future cognitive and mental traits, since head and brain size are positively related to better cognitive function [22,23]. These authors suggest a direct effect on brain development of metformin, which is known to be able to reach the fetus [24,25] and to cross the brain-blood barrier [26], but further specific studies are necessary.

The assessment of the body shape at birth in the present study showed a longer body in the metformin-treated neonates and demonstrated sex-related differences in the response of the offspring to the treatment. In this regard, there were no differences between male counterparts, but metformin-treated females showed a smaller corpulence with smaller thoracic and abdominal circumferences than their female control counterparts. At birth, the abdominal circumference is considered a reliable predictor, with a direct relationship, for the developmental potential during the early postnatal period [17]. Such smaller corpulence of treated females remained during the lactation period and at weaning, but there was also a significant lower corpulence at weaning in the treated males. However, at weaning, traits for muscle development and subcutaneous back fat were higher in both female and male metformin-treated piglets. This is opposite to data on abdominal circumferences since higher values of these traits at weaning prove a good growth in the lactation period and are related to better development, and better yields, during the growing phase [27,28,29].

A possible hypothesis, considering similar body mass with higher corpulence in controls and better muscle development in metformin-treated piglets, would be a reduction of fattening in these latter individuals. In fact, in pigs, the thoracic circumference is considered mainly predictive for the amount of carcass fat while abdominal circumference is considered mainly predictive of visceral fat [30,31,32]. The Iberian piglets from food-deprived pregnancies are characterized by a sex-related catch-up growth: females, but no males, have an enhanced growth during lactation [8]. The objective is counteracting IUGR, but such a catch-up growth may be detrimental in case of overnutrition, leading to obesity and associated disorders [33,34,35,36]. Accordingly, Iberian female piglets from underfed pregnancies have increased body weight, corpulence, and adiposity at juvenile periods compared to females from pregnancies with adequate nutrition [37,38]. A subsequent study from our group indicated that metformin treatment in these piglets during the juvenile development induces an improvement in body mass by favoring muscle deposition without fattening [39], so similar effects might be expected during prenatal and early postnatal development. 

However, these hypotheses should be obviously addressed in future studies; moreover, when a previous study in women with PCOS gave a warning about increased body mass indexes (BMI) in metformin-exposed children [22]. Conversely, evidence found in other clinical studies on pregnant women with diabetes or PCOS indicate similar outputs to those from our study. In brief, children exposed to metformin during pregnancy were larger in size and heavier, but there were no negative effects on the total or abdominal body fat percent or on the metabolic measures [40,41,42,43]. Moreover, one of these trials, similarly to ours, demonstrated more subcutaneous fat without increasing overall body fat in metformin-treated individuals during lactation [43].

The assessment of body composition at weaning demonstrated that control females showed a lower relative weight of the carcasses and a higher relative weight of total viscerae than their male littermates. These results support previous findings in our IUGR model [44,45], addressing that Iberian female fetuses favor the development of the viscerae rather than that of the carcass, conversely to male fetuses (i.e.,: females prioritize the growth of vital organs at the expense of the growth of bones and muscles). Evidence indicates that protective strategies to adapt to nutritional scarcity are more evident in the female offspring, similarly to previous data reviewed by Aiken and Ozanne [46]. In the present study, these differences were not found in the metformin-treated group. However, treated piglets (both females and males) demonstrated a higher relative weight of total viscerae than control animals. Therefore, there was a positive effect of the metformin treatment on the offspring developmental competence. 

However, it is important to note that the individual assessment of each one of the main viscerae addressed that despite no differences or even a non-significant trend to increase in metformin-treated females, the metformin-treated males had lower relative weights of main viscerae (lungs, liver, kidneys, pancreas, spleen and adrenal glands) than the control males. We cannot elucidate if this was a statistical effect of the comparison to total viscera weight (higher in metformin-treated males) due to no differences in the absolute weights of each viscera alone, or if there was a redistribution of the growth of the different viscerae, having in mind that other viscerae were comparatively higher (e.g., intestine). However, these results resemble data from previous studies on the effect of a polyphenol supplementation (i.e., hydroxytyrosol) during pregnancy, where the liver was smaller in treated male offspring and even demonstrated a lower energy content than in their male control counterparts [47,48,49]. Swine models have proved to be highly translational to human medicine, as previously explained [5,7,9]. Thus, these results indicate the need for further specific studies on possible deleterious effects of prenatal therapies regulating fetal homeostasis on male offspring development. Such consideration should be extended to treatments counteracting the effects of maternal PCOS and diabetes during pregnancy.

## 4. Material and Methods

### 4.1. Ethics Statement

The study involved a total of 119 piglets which were born from 15 purebred Iberian sows. All these animals were housed at the INIA facilities, which meet local, national, and European requirements for Scientific Procedure Establishments. The experimental procedures were assessed and approved by the INIA Committee of Ethics in Animal Research and the regional competent authority (report PROEX 353/15) and performed according to the Spanish Policy for Animal Protection RD53/2013, which complies with the European Union Directive 2010/63/UE on the care of animals used for research.

### 4.2. Animal Handling and Experimental Procedure

The experimental procedure was adapted from a previous study evaluating the effect of metformin on fetal development [15]. In brief, the sows were inseminated with cooled semen from a purebred Iberian boar after cycle synchronization with altrenogest (Regumate^®^, MSD, Boxmeer, The Netherlands). All the sows were fed with a standard grain-based food diet (dry matter, 89.8%; crude protein, 15.1%; fat, 2.8%; and metabolizable energy, 3.0 Mcal/kg) adjusted to fulfill 100% of individual daily maintenance requirements during the first 35 days of pregnancy and, in order to impose a nutritional challenge and to induce a higher incidence of IUGR processes as previously described [10,11], reduced to fulfill 50% of such requirements from Day 35 of pregnancy up to the day of delivery. On the same Day 35 of pregnancy, the sows were distributed according to body weight in an untreated control group (group C; *n* = 6) and a group treated with 850 mg of metformin per animal and day (Dianben^®^; Merck Serono, Madrid, Spain), by individually top-dressing over the morning feed from Day 35 to the day of sampling (group METF; *n* = 9). All sows were again fed with the control diet from the day of delivery up to weaning.

### 4.3. Assessment of Neonatal Features and Early Postnatal Development of Piglets

At birth, the total number of piglets was recorded for each sow. Sex, weight, and head and body measurements (biparietal diameter, occipito-nasal length, trunk length, and abdominal and thoracic circumferences) were recorded for each piglet. Head measurements were used to calculate the ratio occipito-nasal length/biparietal diameter. Immediately, all living piglets were tagged with earrings for their identification and underwent within-group fostering to equalize the number of animals among dams. Piglets remained with sows in individual pens until weaning at 30 days old.

All the piglets were again weighted and measured at 15 and 30 days old. Weight values were used to determine the ADWG and the FGR; weight gained per day per starting weight) for the time-intervals. At 30 days old, all the piglets were euthanized by stunning and exsanguination in compliance with standard procedures (RD 53/2013) and sampled for determining the effects of maternal metformin treatment on early postnatal body weight and size, adiposity, and body composition. The head was immediately separated from the trunk at the atlanto-occipital joint and weighted, all thoracic and abdominal viscerae were removed, and the weight of the carcass and the total viscerae together were recorded. The back-fat depth and the loin diameter were recorded with a measuring tape at the carcass, and the major organs (heart, lungs, liver, intestine, kidney, spleen, pancreas, and adrenal glands) were weighed individually. The following weight ratios were considered: weights of head to body weight and weights of brain, heart, lungs, liver, kidneys, intestine, pancreas, spleen, and adrenal glands relative to both total body weight and total viscera weight. 

### 4.4. Statistical Analyses

Data were analyzed using SPSS 22.0 (IBM, NY). T-student tests were used to assess the effects of maternal treatment (control vs. treated) on litter size and distribution of sexes. Effects of treatment (control vs. treated) and sex (female vs. male) on changes over time in weight and measures were assessed by ANOVA for repeated measures with the Green–Houser–Geisser correction when statistically significant while differences in adiposity, loin diameter, and organs weights were assessed by two-way ANOVA. Duncan’s *post-hoc* test was performed to check differences among groups in multiple comparisons. All results were expressed as mean ± SEM, and statistical significance was accepted from *p* < 0.05.

## 5. Conclusions

Overall, the results obtained in the present study show the efficacy of metformin to improve the developmental competence of fetuses from compromised pregnancies. However, there is also evidence of a prominent role of the offspring sex on its response to maternal metformin therapies, which may be even deleterious in the case of male offspring. Such evidence supports the concept of personalized medicine including offspring sex in the case of pregnancies and, specifically, should be taken in account for further preclinical studies and even current clinical application in pregnant women affected by PCOS and diabetes.

## Figures and Tables

**Table 1:** Mean (± SEM) values for birthweight and size in newborn piglets from controls (group C) and sows treated with metformin during pregnancy (group METF).

Parameter	Group C		Group METF	
	Females	Males	Females	Males
Body weight (g)	1.13 ± 0.03	1.15 ± 0.03	1.11 ± 0.03	1.16 ± 0.03
Body length (cm)	23.27 ± 0.34 ^a^	23.03 ± 0.28 ^e^	23.72 ± 0.30 ^b^	23.92 ± 0.29 ^f^
Occipito-nasal length (cm)	11.14 ± 0.22 ^e^	11.31 ± 0.19 ^e^	11.93 ± 0.13 ^f,1^	12.27 ± 0.16 ^f,2^
Biparietal diameter (cm)	4.57 ± 0.08 ^a^	4.58 ± 0.07 ^a^	4.77 ± 0.05 ^b^	4.77 ± 0.07 ^b^
Thoracic circumference (cm)	23.25 ± 0.29 ^c^	22.48 ± 0.29	22.20 ± 0.19 ^d,1^	22.64 ± 0.27 ^2^
Abdominal circumference (cm)	18.76 ± 0.31 ^a^	18.63 ± 0.30	17.83 ± 0.23 ^b^	18.28 ± 0.30

Superscripts denote significant differences between treatments (a ≠ b: *p* < 0.05; c ≠ d: *p* < 0.005; e ≠ f: *p* < 0.0005) and between sexes within treatments (1 ≠ 2: *p* < 0.05).

**Table 2:** Mean (±SEM) values for absolute weight of different organs and structures at weaning (i.e., 30 days old) in offspring from control (group C) and sows treated with metformin during pregnancy (group METF).

Parameter (g)	Group C		Group METF	
	Females	Males	Females	Males
Body	4986.76 ± 112.65	4895.59 ± 137.63	4728.75 ± 74.11	4777.13 ± 88.72
Head	689.67 ± 9.23	666.71 ± 12.68	653.42 ± 10.45	645.22 ± 6.99
Carcass	3053.83 ± 51.04	3219.43 ± 98.94	3048.53 ± 110.45	2955.56 ± 104.3
Total viscerae	835.00 ± 17.76	755.29 ± 24.49	895.0 ± 16.88	849.59 ± 16.64
Heart	32.17 ± 0.50	30.57 ± 0.62	35.38 ± 0.77	35.09 ± 1.08
Lungs	71.67 ± 1.65	81.14 ± 3.21	79.66 ± 1.47	77.44 ± 1.36
Liver	131.00 ± 1.63	131.86 ± 3.11	132.10 ± 2.62	131.56 ± 2.52
Intestine	429.17 ± 10.80	352.86 ± 17.31	439.72 ± 9.70	411.48 ± 7.88
Kidneys	28.33 ± 0.38	29.71 ± 0.60 ^a^	28.36 ± 0.47	27.00 ± 0.43 ^b^
Spleen	11.42 ± 0.18	16.39 ± 0.67	15.45 ± 0.50	13.95 ± 0.52
Pancreas	4.99 ± 0.28	6.11 ± 0.13	5.57 ± 0.20	5.32 ± 0.18
Adrenal glands	0.71 ± 0.02	0.95 ± 0.02	0.85 ± 0.02	0.88 ± 0.02

Superscripts denote significant differences between treatments (a ≠ b: *p* < 0.05).
